# Protective Effect of N-Arachidonoyl Glycine-GPR18 Signaling after Excitotoxical Lesion in Murine Organotypic Hippocampal Slice Cultures

**DOI:** 10.3390/ijms20061266

**Published:** 2019-03-13

**Authors:** Urszula Grabiec, Tim Hohmann, Chalid Ghadban, Candy Rothgänger, Daniel Wong, Alexandra Antonietti, Thomas Groth, Ken Mackie, Faramarz Dehghani

**Affiliations:** 1 Institute of Anatomy and Cell Biology, Medical Faculty of Martin Luther University Halle-Wittenberg, Grosse Steinstrasse 52, 06108 Halle (Saale), Germany; urszula.grabiec@medizin.uni-halle.de (U.G.); tim.hohmann@medizin.uni-halle.de (T.H.); chalid.ghadban@medizin.uni-halle.de (C.G.); candy.rothgaenger@medizin.uni-halle.de (C.R.); deagdeag@gmail.com (D.W.); alexandra_antonietti@yahoo.de (A.A.); 2 Biomedical Materials Group, Institute of Pharmacy & Interdisciplinary Center for Materials Science, Martin Luther University Halle-Wittenberg, Heinrich-Damerow Strasse 4, 06120 Halle (Saale), Germany; thomas.groth@pharmazie.uni-halle.de; 3 Department of Psychological & Brain Sciences, Indiana University, 1101 E. 10th, Bloomington, IN 47405, USA; kmackie@indiana.edu

**Keywords:** microglia, neuroprotection, N-arachidonoyl glycine

## Abstract

N-arachidonoyl glycine (NAGly) is an endocannabinoid involved in the regulation of different immune cells. It was shown to activate the GPR18 receptor, which was postulated to switch macrophages from cytotoxic to reparative. To study GPR18 expression and neuroprotection after NAGly treatment we used excitotoxically lesioned organotypic hippocampal slice cultures (OHSC). The effect of NAGly was also tested in isolated microglia and astrocytes as these cells play a crucial role during neuronal injury. In the present study, the GPR18 receptor was found in OHSC at mRNA level and was downregulated after N-Methyl-D-aspartate (NMDA) treatment at a single time point. Furthermore, treatment with NAGly reduced neuronal damage and this effect was abolished by GPR18 and cannabinoid receptor (CB)_2_ receptor antagonists. The activation but not motility of primary microglia and astrocytes was influenced when incubated with NAGly. However, NAGly alone reduced the phosphorylation of Akt but no changes in activation of the p44/42 and p38 MAPK and CREB pathways in BV2 cells could be observed. Given NAGly mediated actions we speculate that GPR18 and its ligand NAGly are modulators of glial and neuronal cells during neuronal damage.

## 1. Introduction

Neuroprotective effects have been reported for several cannabinoids in different models. N-arachidonoyl glycine (NAGly) is a product of the oxidative metabolism of anandamide and shares a structural similarity with this endocannabinoid [1,2,3]. NAGly is believed to activate the cannabinoid receptor GPR18 with no affinity for cannabinoid receptor (CB)_1_ [1,2,3] and transient receptor potential vanilloid 1 (TRPV1) [1]. About effects of NAGly on CB_2_, which is expressed in the central nervous system and on the immune cells, little is known [4].

GPR18 is a seven-transmembrane G-protein coupled receptor consisting of 331 amino acids. GPR18 has been found in peripheral blood cells, lymphoid tissues, macrophages with different expression levels for cytotoxic and reparative cells [5]. It is also present in the brain [6,7] and in some glioblastoma multiforme cells [8]. gpr18 expression in murine BV2 microglia was shown by McHugh [9] and questioned by Finlay [8]. After application of NAGly (10 µM) to GPR18 transfected cell lines a calcium mobilization and pertussis toxin-sensitive inhibition of adenylyl cyclase were observed [7], indicating that NAGly is an endogenous ligand acting via a G_αi/o_ protein dependent mechanism. In the rat model of inflammatory pain NAGly reduced the mechanical allodynia and thermal hyperalgesia in a CB_1_ and CB_2_ independent way [10]. Since GPR18 was highly expressed on the cellular components of the immune system, different effects of NAGly on immune cells were examined. When given orally, NAGly reduced the migration of proinflammatory cell types into the peritoneal cavity [11]. Furthermore, NAGly induced migration of BV2 microglia in Boyden chamber assays [9,12] (for review Burstein 2014). Takenouchi et al. 2012 showed that NAGly caused apoptosis in the macrophage-derived cell line RAW264.7 [5]. NAGly as a putative GPR18 ligand is controversially discussed in the literature [8,13,14].

The present work was designed to analyze the impact of GPR18 and its ligand NAGly on neuronal injury. The expression of GPR18 was examined in brain tissue, glial and neural derived cells. To study the neuroprotective properties of NAGly an established and very well-characterized in vitro model of organotypic hippocampal slice cultures (OHSC) mimicking in vivo conditions was used [15,16,17,18]. Using N-Methyl-D-aspartate (NMDA) treated OHSC the changes in gpr18 expression after the lesion were assessed. Furthermore, the protective effects of NAGly on excitotoxically damaged OHSC were tested and the potential mechanisms investigated using GPR18 and CB_2_ antagonists. Using isolated microglia and astrocytes, the effects of NAGly in terms of morphology and function were examined as well as the activation of signaling pathways associated with cell survival in glial cells. 

## 2. Results

### 2.1. GPR18 mRNA and Protein are Expressed in Primary Astrocytes, Microglia and Neurons

**Figure 1:**
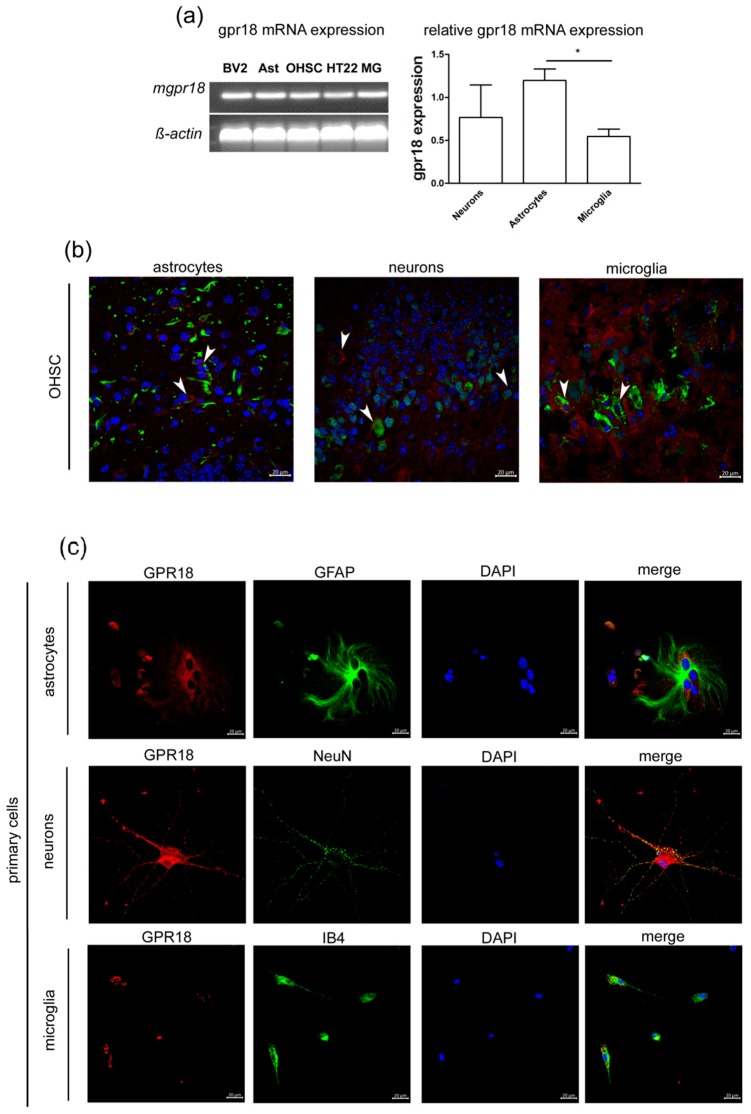
Murine microglia, astrocytes, organotypic slices cultures and cell lines BV2, neuronal cell line HT22 express mRNA for GPR18. (**a**) The gpr18 mRNA was detected in BV2 microglia (BV2), primary microglia (MG), astrocytes (Ast), organotypic hippocampal slice cultures (OHSC) and hippocampal neuronal cell line HT22. The relative concentrations of mRNA were determined via qRT-PCR and normalized to ß-actin. Primary hippocampal neurons (*n* = 3) express gpr18 mRNA, astrocytes (*n* = 3) express significantly more gpr18 mRNA than microglia (*n* = 6, *p* < 0.05). Expression of GPR18 receptor in (**b**) OHSC and (**c**) primary cells. (**b**) OHSC: GPR18 (red) was found to be colocalized with GFAP (green) in murine astrocytes, NeuN (green) in primary hippocampal neurons and IB4 (green) in microglia (arrows). (**c**) Primary astrocytes, hippocampal neurons and microglia express GPR18 protein. DAPI (blue) was used to stain DNA in nuclei. Scale bar = 20 µm. The asterisk denotes significant results regarding the respective measurement indicated with the bar.

GPR18 was found on astrocytes (GFAP), neurons (NeuN) and microglia (IB4) (Figure 1). The quantitative analyses of gpr18 mRNA expression levels have shown highest values in astrocytes (1.2 ± 0.13) followed by primary hippocampal neurons (0.77 ± 0.37) and microglia (0.54 ± 0.09). The difference between astrocytes and microglia was statistically significant (*p* < 0.05) (Figure 1a). We detected the expression of the GPR18 receptor in the microglial cell line BV2, primary astrocytes, OHSC, primary microglia and the hippocampal neuronal cell line HT22 (Figure 1a). 

### 2.2. Slight Changes in gpr18 mRNA and No Changes in GPR18 Protein Expression in OHSC

**Figure 2:**
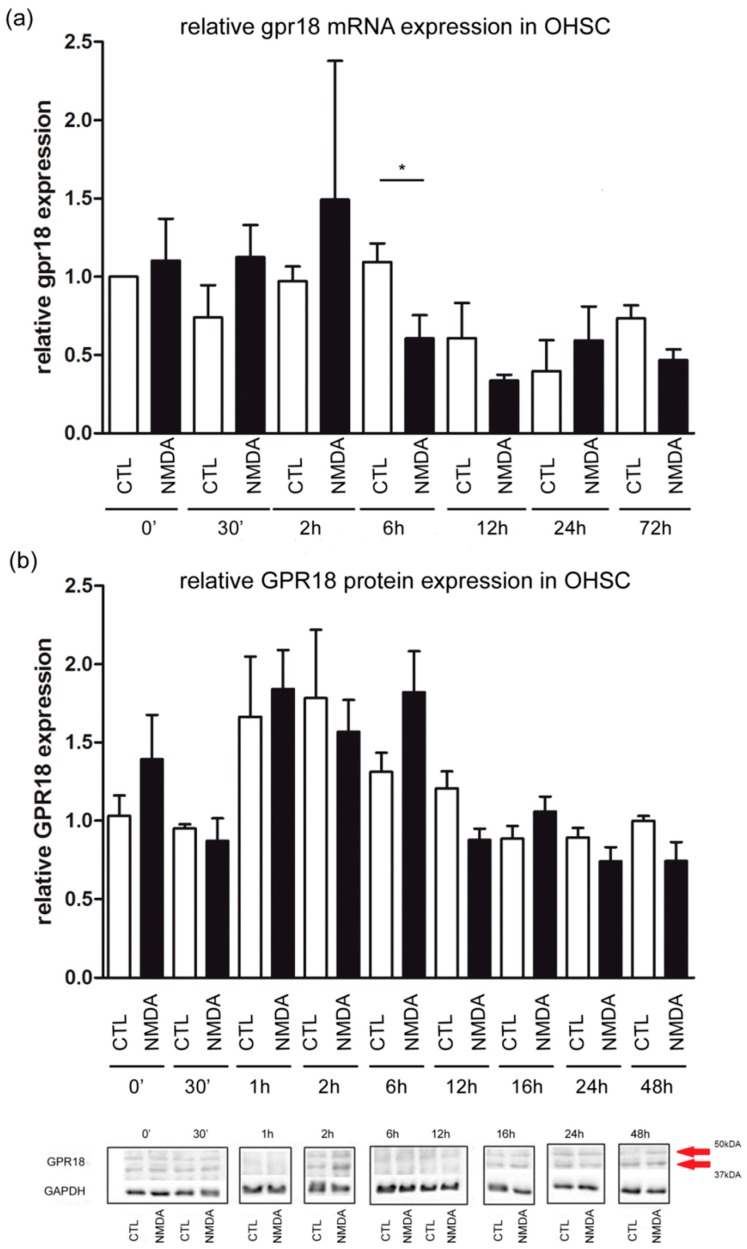
GPR18 expression measured over time. (**a**) The relative concentrations of mRNA were determined by qRT-PCR at time points 0 h, 30 min, 2 h, 6 h, 12 h, 24 h and 72 h. Cycle thresholds were normalized to ß-actin. The ΔCt of the control group at time 0 was used for quantification. Each value is presented as the mean (±SEM) of at least 3 replicates, each replicate contains 2 to 3 OHSCs (n_CTL0h_ = 3; n_NMDA0h_ = 3; n_CTL30′_ = 3; n_NMDA30′_ = 4; n_CTL2h_ = 4; n_NMDA2h_ = 4; n_CTL12h_ = 4; n_NMDA12h_ = 3; n_CTL24h_ = 3; n_NMDA24h_ = 3; n_CTL72h_ = 4; n_NMDA72h_ = 4). After 6 h of excitotoxical lesion gpr18 level decreased significantly (CTL: 1.09 ± 0.11, n_CTL6h_ = 4; NMDA: 0.6 ± 0.22, n_NMDA6h_ = 5; NMDA 6 h vs. CTL 6 h *p* < 0.05). (**b**) Changes in the expression of the GPR18 protein (arrows) over 48 h after NMDA treatment in OHSC at time points 0 h, 30 min, 1 h, 2 h, 6 h, 12 h, 16 h, 24 h and 48 h assessed in Western blot analysis. No significant changes over time after NMDA treatment were observed (n_CTL0h_ = 6; n_NMDA0h_ = 5; n_CTL30′_ = 4; n_NMDA30′_ = 3; n_CTL1h_ = 5; n_NMDA1h_ = 5; n_CTL2h_ = 4; n_NMDA2h_ = 6; n_NMDA6h_ = 7; n_NMDA6h_ = 10; n_CTL12h_ = 3; n_NMDA12h_ = 3; n_CTL24h_ = 5; n_NMDA24h_ = 6; n_CTL48h_ = 5; n_NMDA48h_ = 5). Furthermore, representative Western blot for all time points performed on different membranes are shown. GAPDH was used as house-keeping protein. The asterisk denotes significant results regarding the respective measurement indicated with the bar.

No significant effects were observed for time points 30 min, 2 h, 12 h, 24 h and 72 h in mRNA expression in comparison to the control group. The relative expression of gpr18 in OHSC was measured. After 6 h of excitotoxical lesioning gpr18 levels decreased significantly (CTL: 1.09 ± 0.11; NMDA: 0.6 ± 0.22, NMDA 6 h vs. CTL 6 h *p* < 0.05, Figure 2a). 

The specificity of the antibody was tested by the use of a blocking peptide as published before [9]. The expression of GPR18, normalized to GAPDH and to the respective time control in OHSC did not significantly change after 30 min, 1 h, 2 h, 6 h, 12 h, 16 h, 24 h and 48 h in comparison to the control group (Figure 2b).

### 2.3. NAGly is Neuroprotective in NMDA Lesioned OHSC

**Figure 3:**
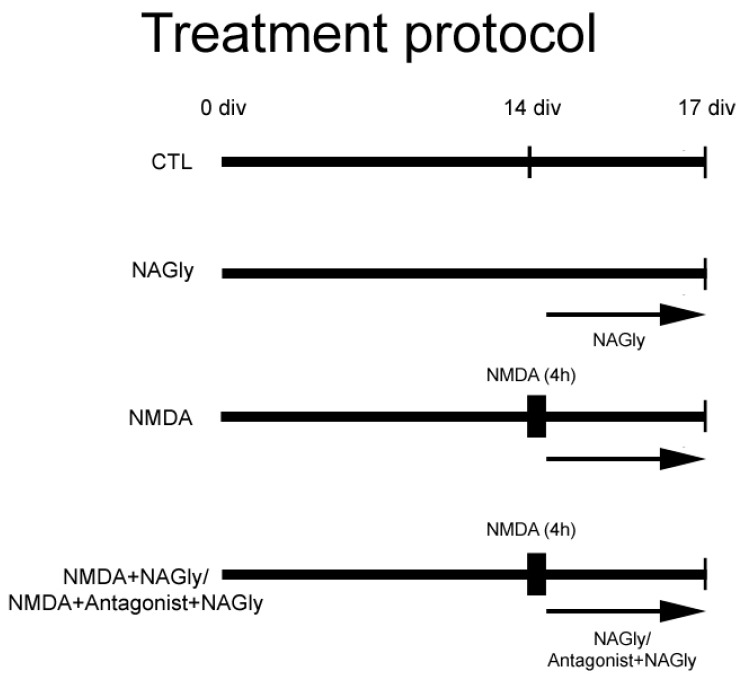
Treatment protocol. Some OHSC were kept in culture medium and served as controls (CTL). A further group was lesioned with NMDA and conduced as a positive control (NMDA). Cannabinoids were applied alone on day 14 to OHSC (NAGly) or following 4 h incubation with NMDA (NMDA + NAGly). Some OHSC were pre-incubated with an antagonist for CB_2_ (AM630) or GPR18 (O-1918) for 15 min before adding NAGly. The fixation was performed on Day 17.

The treatment of OHSC was performed according to treatment protocol (Figure 3).

**Figure 4:**
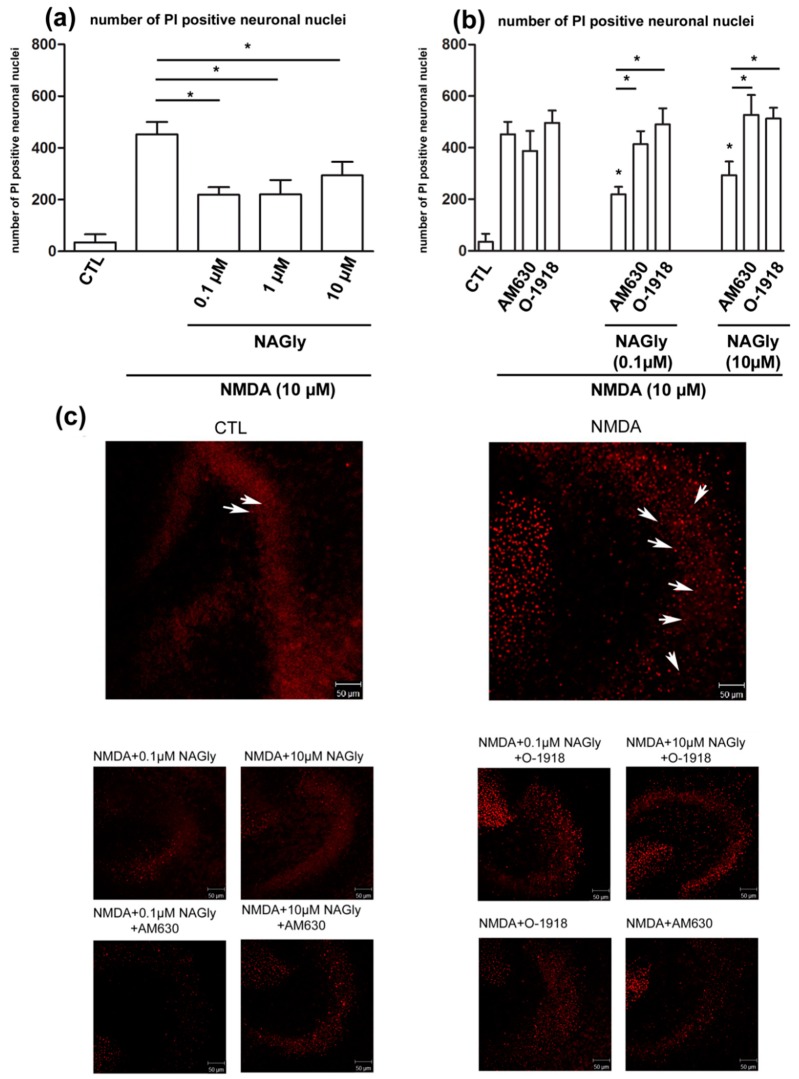
NAGly’s mediated neuroprotection. (**a**) NAGly (0.1 µM, 1 µM, 10 µM) is protective in organotypic hippocampal slice culture after NMDA (10 µM) damage. In the control group few PI (arrows) positive dead neurons were quantified in the region of dentate gyrus. Application of NMDA (4 h) led to excitotoxical damage in OHSC and was prevented by following incubation with NAGly for 72 h. The increase in the number of dead neurons was significantly reversed by incubation with NAGly (n_CTL_ = 17; n_NAGly_ = 5; n_NMDA_ = 28; n_NMDA+NAGly0.1µM_ = 19; n_NMDA+NAGly1µM_ = 6; n_NMDA+NAGly10µM_ = 15). (**b**) Application of antagonists, O-1918 (30 µM) or AM630 (10 µM) to NMDA lesioned OHSC had no impact on the amount of damaged cells (n_CTL_ = 17; n_NMDA_ = 28; n_NMDA+AM630_ = 5; n_NMDA+O-1918_ = 10). The neuroprotective effects of NAGly (0.1 µM and 10 µM) were effectively blocked using GPR18 antagonist, O-1918 and CB_2_ antagonist, AM630 (n_NMDA+NAGly0.1µM_ = 19; n_NMDA+NAGly10µM_ = 15; n_NMDA+NAGly0.1µM+O-1918_ = 10; n_NMDA+NAGly10µM+O-1918_ = 11; n_NMDA+NAGly0.1µM+AM630_ = 10; n_NMDA+NAGly10µM+AM630_ = 6). (**c**) Representative pictures of OHSC. The damage in dentate gyrus and the effects of cannabinoids were assessed. In the control group very few PI positive cells are observed. In contrast, incubation with NMDA led to massive increase in amount of damaged neurons. NAGly reduced the number of PI positive cells; this effect was attenuated by co-application with AM630 or O-1918. Scale bar = 50 µm. The asterisk denotes significant results regarding the respective measurement indicated with the bar.

In control slices (34.82 ± 31.81) only a few damaged neurons were observed. Application of NMDA led to a strong increase in the number of propidium iodide (PI) positive neurons (452.40 ± 48.13; Figure 4a).

NAGly (NMDA + 0.1 µM NAGly: 218.90 ± 29.57; NMDA+1 µM NAGly: 220.30 ± 55.50; NMDA + 10 µM NAGly: 293.70 ± 52.63, for all *p* < 0.05 vs. NMDA *p* < 0.05) significantly reduced the number of PI positive neurons compared to NMDA treated slice cultures in all (0.1 µM, 1 µM, 10 µM) used concentrations (Figure 4a).

### 2.4. NAGly Mediated Neuroprotection is Possibly Mediated by GPR18 and CB_2_ Receptors in OHSC

Application of AM630 (NMDA + AM630: 387.8 ± 77.45) or O-1918 (NMDA + O-1918: 496 ± 48.43) to excitotoxically damaged OHSC had no effect on the number of PI positive cells (Figure 4b). If NAGly (0.1 µM or 10µM) was co-applied with AM630 (NMDA + 0.1 µM NAGly + AM630: 413.9 ± 50.34; NMDA + 10 µM NAGly + AM630: 527.7 ± 76.37; for all *p* < 0.05 vs. NMDA + NAGly 0.1 µM or 10 µM *p* < 0.05) or O-1918 (NMDA + 0.1 µM NAGly + O.1918: 490.1 ± 62.79; NMDA + 10 µM NAGly + O-1918: 513.3 ± 41.62; for all *p* < 0.05 vs. NMDA + NAGly 0.1 µM or 10 µM *p* < 0.05) the neuroprotective effect was missing, indicating GPR18 and CB_2_ receptor dependent actions (Figure 4b).

### 2.5. Ramification Index of Isolated Primary Microglia

**Figure 5:**
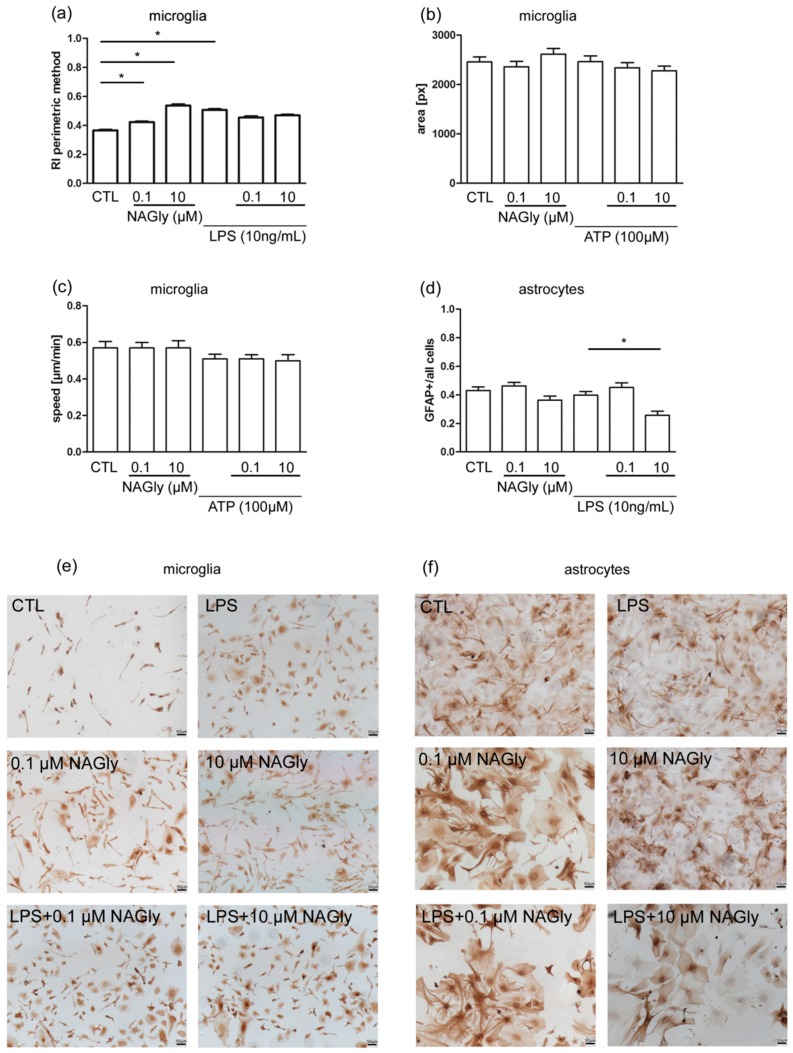
Mechanism behind NAGly mediated neuroprotection in primary microglia (**a**–**c**) and primary astrocytes (**d**). (**a**) Incubation with NAGly (1 µM; n_NAGly0.1µM_ = 2281) in microglia led to a 15% significant increase in ramification index in comparison to control (n_CTL_ = 2607). Higher concentration of NAGly (10 µM; n_NAGly10µM_ = 1253) showed a stronger rise of 46%. After incubation with LPS (n_LPS_ = 2021) in combination with NAGly (0.1 µM; n_LPS+NAGly0.1µM_ = 1002, 10 µM, _nLPS+NAGly10µM_ = 1628) no significant effects were observed. The ramification index is approximately 1 for amoeboid cells and tends to 0 for strongly ramified cells. (**b**) The area and (**c**) speed of primary wild type microglia cells were not altered after treatment with NAGly or ATP or both. The number of analyzed cells is the same for area and speed (n_CTL_ = 112; n_NAGly0.1µM_ = 114; n_NAGly10µM_ = 105, n_ATP_ = 128; n_ATP+NAGly0.1µM_ = 122; n_ATP+NAGly10µM_ = 90). (d) NAGly (n_0.1µM_ = 85; n_10µM_ = 55) alone did not change the level of GFAP expression significantly versus CTL (n_CTL_ = 87) in primary astrocytes. Treatment with LPS (10 ng/mL; n_LPS_ = 65) alone did not increase the activation of astrocytes measured as GFAP index. GFAP index was defined as ratio of GFAP positive astrocytes to all astrocytes. Treatment with LPS and NAGly in low concentration (n_LPS+NAGly0.1µM_ = 55) had no effect on the astrocytes activation. NAGly in high concentration (0.26 ± 0.03, n_LPS+NAGly10µM_ = 35; *p* < 0.05 vs. LPS) significantly reduced the GFAP positive ratio in comparison to LPS alone. (**e**) Representative pictures of primary microglia stained with IB4. (**f**) Representative images of astrocytes stained with GFAP. Scale bar = 50 µm. The asterisk denotes significant results regarding the respective measurement indicated with the bar.

Immunohistochemical staining and the following analysis of microglia stimulated for 48 h were used to determine the ramification index. Primary microglia cells incubated in medium alone were used as control (CTL: 0.37 ± 0.23). Treatment with NAGly (0.1 µM, 10 µM) alone induced amoeboid morphology reflected by an increase in the ramification index (0.1 µM NAGly: 0.42 ± 0.26, *p* < 0.05 vs. CTL; 10 µM NAGly:0.54 ± 0.29, *p* < 0.05 vs. CTL). Application of LPS (10 ng/mL) induced an amoeboid morphology of microglia (LPS: 0.51 ± 0.23, *p* < 0.05 vs. CTL). Co-application of LPS and NAGly (0.1 µM or 10 µM) did not change the ramification index (LPS + 0.1 µM NAGly: 0.46 ± 0.24, *p* > 0.05 vs. LPS; LPS + 10 µM: 0.47 ± 0.21, *p* > 0.05 vs. LPS) (Figure 5a,e).

### 2.6. NAGly Did Not Induce Changes in the Motility of Isolated Wild Type Microglia

Using live cell imaging, microglia were analyzed over 24 h. Mean area and mean speed over time were measured for the same cells. Treatment with ATP (100 µM: speed: 0.51 ± 0.25 µm/min, area: 2466 ± 1314 px) or NAGly (1 µM: speed: 0.57 ± 0.32 µm/min, area: 2362 ± 1138 px; 10 µM: speed: 0.57 ± 0.4 µm/min, area: 2616 ± 1176 px) or combination of both (ATP + NAGly 0.1 µM: speed: 0.51 ± 0.25 µm/min, area: 2341 ± 1138 px; ATP + NAGly 10 µM: speed: 0.5 ± 0.31 µm/min, area: 2279 ± 902 px) did not significantly affect the size or speed of the microglia in comparison to the control (speed: 0.57 ± 0.37 µm/min, area: 2459 ± 1076 px) (Figure 5b,c). 

### 2.7. Activation of Cultured Astrocytes Measured as GFAP Index

The activation of astrocytes is correlated with the level of GFAP expression. Astrocytes in culture medium were set as control (CTL: 0.43 ± 0.02). Incubation with NAGly (0.1 µM or 10 µM) or LPS (10 ng/mL) alone did not lead to a significant change (0.1 µM NAGly: 046 ± 0.22, *p* > 0.05 vs. CTL; 10 µM NAGly: 0.36 ± 0.03, *p* > 0.05; LPS: 0.39 ± 0.02, *p* > 0.05) in comparison to the control group. 

Treatment with LPS and 0.1 µM NAGly did not affect astrocyte activation in comparison to control group (LPS + 0.1 µM NAGly: 0.45 ± 0.03, *p* > 0.05). After co-incubation with LPS and 10 µM NAGly the GFAP index was significantly reduced if compared to LPS group (LPS + 10 µM NAGly: 0.26 ± 0.03; *p* < 0.05) (Figure 5d,f).

### 2.8. Activation of 44/42 MAPK and pCREB is Not Altered, Whereas pAkt is Downregulated Only at 30 min in NAGly Treated BV2 Cells

**Figure 6:**
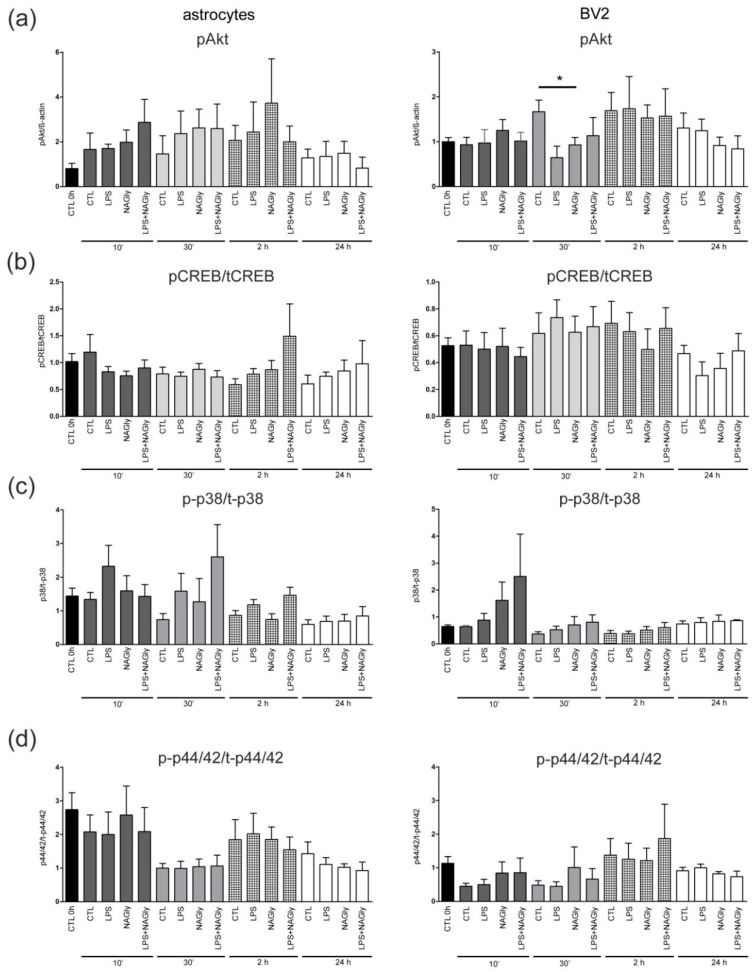
NAGly (0.1 µM) and intracellular signaling cascades analyzed using Western blot. The BV2 cells and primary astrocytes (*n* > 3) were collected after treatment with NAGly and/or LPS (10 ng/mL) for 10 min, 30 min, 2 h and 24 h and screened for activation of signaling cascades (**a**) Akt, (**b**) CREB, (**c**) p38 MAPK or (**d**) p44/42 MAPK. (**a**) NAGly led to decrease in pAkt after 30 min in comparison to the control group (CTL 30 min) in BV2 cells (n_0h_ = 47; n_CTL10′_ = 16; n_NAGly10′_ = 11; n_CTL30′_ = 15; n_NAGly30′_ = 11; n_CTL2h_ = 12; n_NAGly2h_ = 9; n_CTL6h_ = 12; n_NAGly6h_ = 9; n_CTL24h_ = 14; n_NAGly24h_ = 10). (**b**–**d**) In the BV2 cells or astrocytes pCREB, p-p38 and p-p44/42 were not affected by NAGly. The asterisk denotes significant results regarding the respective measurement indicated with the bar.

**Figure A1:**
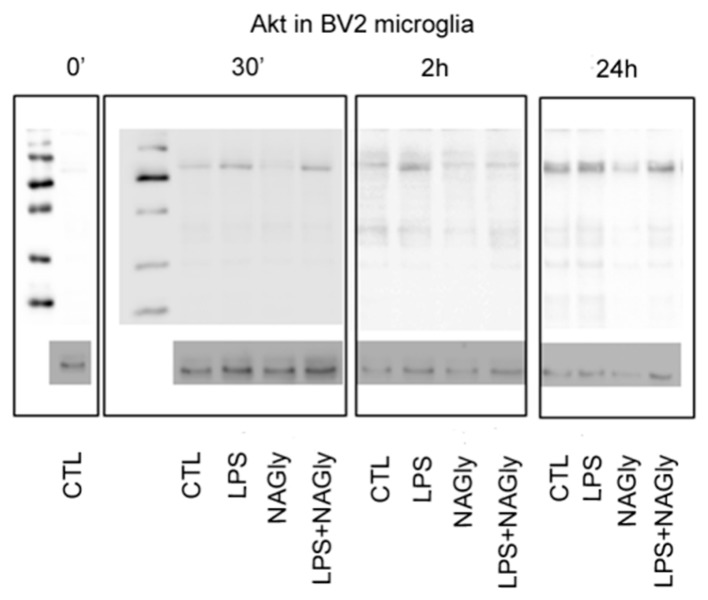
Representative Western blot membrane of Akt (60 kDa) after 30 min, 2 h and 24 h in BV2 cells. For better visualization contrast and brightness were enhanced. Western blots were performed on different membranes; ß-actin was used as a house-keeping protein.

To understand underlying mechanisms of NAGly-induced effects in primary astrocytes and BV2 microglia cell line, we focused on the downstream signaling pathways including Akt, CREB, p38 and p44/42 MAPK. NAGly alone or in combination with 10 ng/mL LPS did not significantly affect the amount of pCREB, p-p38 and p-p44/42 MAPK after the indicated incubation time alone (Figure 6). Interestingly, NAGly alone significantly reduced (*p* < 0.05) pAkt after 30 min in comparison to corresponding control (Figure 6, Figure A1).

## 3. Discussion

Neuronal injuries are a major cause of death and hospitalizations [19] although intrinsic protective systems are present in the brain and spinal cord. Cannabinoids are capable of inducing neuroprotective effects during and after the neuronal injury and the regulation of their levels is considered as a potential target in therapy [20,21,22,23]. NAGly was identified as an endogenous cannabinoid which activates the GPR18 receptor and modulates the immune system [7,11]. In an earlier study the presence of GPR18 was shown on microglia cells and its role in microglia migration and cytokine production was postulated [12,24]. 

In this study mRNA for GPR18 was found in microglia, microglia cell line BV2, astrocytes, primary neurons, neuron like cell line HT22 and in OHSC. Evaluation using immunohistochemistry confirmed protein expression in primary astrocytes, hippocampal neurons and microglia. We analyzed the dynamics of GPR18 receptor expression after excitotoxic neuronal injury. In contrast to unlesioned OHSC, gpr18 mRNA expression but not protein was temporarily reduced in NMDA lesioned slices. NMDA seems to have little or no effect on GPR18 mRNA expression except for single time point (6 h) no significant changes were observed. At protein level the amount of GPR18 was not different between NMDA and CTL groups at all corresponding time points level. Possible explanation for missing correlation between mRNA and protein might be the fact that expression of a gene and its translation into protein are timely and spatially not in close correlation. In an organotypic model like OHSC the detected values for mRNA or protein are the result of changes in all cell types. The expression of gpr18 mRNA has been reported to be markedly increased in proinflammatory stimulated murine peripheral macrophages [5]. This discrepancy might be explained by the fact that (a) in OHSC, a blood flow and subsequently an infiltration of immune cells does not occur and peripheral macrophages/monocytes are mostly absent and (b) an upregulation of gpr18 in microglia cells might be accompanied by a downregulation of receptor expression in neurons and astrocytes. The sum of receptor amount would therefore decrease despite an activation state of brain immune cells after lesion. Interestingly, in our previous results and in recent studies an activation and proliferation of microglia was associated with neuronal protection against excitotoxicity [25,26,27,28,29]. 

The GPR18 ligand NAGly was found at first in the spinal cord, small intestine and brain [1] and it seems to be less abundant in the tissues with high GPR18 levels [1,6,30]. It should be taken into consideration that endocannabinoids are produced on demand, and their fast metabolism complicates their verification [23]. By adding exogenous ligands like NAGly to OHSC treated with NMDA, specific processes during and after traumatic brain injury could be mimicked [31], secondary injury reduced and intrinsic effects on damaged brain tissue observed. NAGly (0.1 µM, 1 µM, 10 µM) reduced the neuronal damage in excitotoxically lesioned OHSC. Since NAGly was applied 4 h after initial injury, a complete preservation of tissue cannot be achieved. Previously, other cannabinoids such as N-arachidonoyl dopamine (100 pM to 10 µM), 2-arachidonoyl glycerol (0.001–1 µM) and palmitylethanolamide (0.01–1 µM) were shown to exert neuroprotection in OHSC in a wide range of concentration (from pM to µM) in the same and further models [17,20,21,22,23,32,33,34]. This is in agreement with here observed neuroprotective effects of NAGly over three magnitudes of concentrations in excitotoxically lesioned OHSC without dose dependency in this concentration range. Since this data are obtained in vitro further evaluation in in vivo models is recommendable. Furthermore, increasing endocannabinoid levels by blockade of metabolizing enzymes like MAGL protective after neuronal damage [35]. The neuroprotective effect of NAGly was abolished after blockade of the GPR18 or CB_2_ receptor. NAGly was shown before to inhibit T lymphocyte proliferation partly via CB_2_ [36]. Additionally, GPR18 and CB_2_ were shown recently to be co-expressed and form heterodimers in microglia [37]. The interaction between CB_2_ and GPR18 receptors led to negative cross talk and cross-antagonism, since GPR18 antagonism prevented effects mediated by CB_2_ agonist [37]. Therefore, it is plausible that the blockade of NAGly effects by both the CB_2_ and GPR18 antagonists observed here is an effect of cross-antagonisation. Similar to our study, Franklin and Stella suggested that both receptors, CB_2_ (blocked by AM630) and abnormal Cannabidiol receptors (blocked by O-1918) regulate microglia actions [38]. 

**Figure A2:**
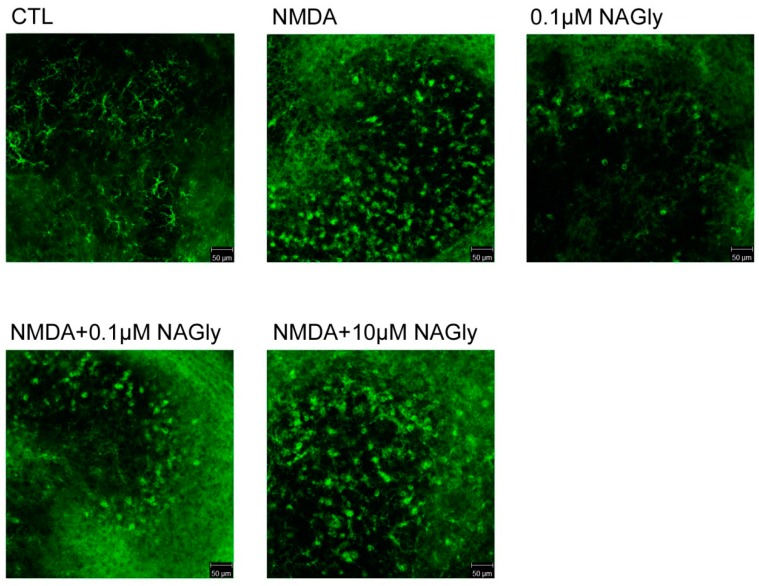
In green IB4-positive microglia are shown in the dentate gyrus of OHSC. CTL: In control medium microglia cells showed in dentate gyrus a ramified morphology with extensive branching of cytoplasmic processes. After NMDA (10 µM) lesion microglial cells become amoeboid and their number increased. Microglia displayed a ramified morphology after incubation with 0.1 µM NAGly alone. Treatment with NMDA and NAGly (0.1 µM, 10 µM) led to amoeboid morphology of microglial cells and an increase in their numbers. Qualitatively no obvious differences in the morphology of microglia was found when NAGly treated groups were compared to those without NAGly. Scale bar = 50 µm.

The morphology of microglial cells plays an important role during neuronal damage and was changed toward a more amoeboid morphology by NAGly reflected by an increase in ramification index, and in OHSC microglia which showed after incubation with NAGly alone an amoeboid morphology (Figure A2). In the amoeboid form, microglial cells have been shown to migrate through the brain tissue to the lesion [39]. Changes in microglia morphology were reported to be mediated by TLR4 receptor after activation with LPS [40]. Endocannabinoids can affect cell migration and motility in different cell types [41,42]. NAGly and THC are both agonists on GPR18 and they induced migration of human endometrial cells, immortalized microglia cell line BV2, and macrophages [7,9,24,43]. The missing effect of NAGly on motility may be explained by the assay used in this study. Till now only changes in chemotaxis measured by Boyden chamber assay and no direct motility measurements were conducted for cannabinoids and NAGly. Furthermore, previous studies used the microglial cell line BV2 and no primary microglia like in this report [9]. ATP is regularly used as a chemoattractant in experiments with primary microglia cells; their physiology can be distinguished in many aspects from the cell line BV2. In our system, motility of single cells was examined in a uniform culture medium (without treatment) rather than directed migration in a chemotactic ATP gradient, explaining the difference in our results to those obtained by other labs. The comparison between migration assays reveals that by using the Boyden chamber, the number of cells passing through a membrane over a defined period of time (3–6 h) was counted whereas in live cell imaging, single cells were observed over a 24 h period and the mean speed was calculated [12,24,41]. The absence of a concentration gradient is a plausible explanation for the missing induction of increased motility after NAGly or ATP treatment. However, it cannot be excluded that treatment with NAGly results in release of factors which affect microglia motility or directed migration.

Characterization of NAGly induced effects on further glial cells and astrocytes is complex. Astrocytes have a much broader role than merely supporting the neurons in the brain, as they have specialized functions in inducing and regulating the blood brain barrier and signal transduction pathways [44]. Glial fibrillary acidic protein (GFAP) is the main intermediate filament protein in mature astrocytes [44]. The enlargement of astrocytes and the increased expression of GFAP is an indicator of reactive gliosis, a process which has been related to brain damage. In this study we used a GFAP antibody, which exposes cells with stellate morphology, characteristic for differentiated astrocytes. The disadvantage of this staining is that only reactive astrocytes become labeled and therefore not the whole population of astrocytes shows a positive immunoreactivity. NAGly seems to affect the activation of astrocytes under pathological conditions when they are treated with LPS. In our study LPS (10 ng/mL) alone did not induce an increase in GFAP expression in primary astrocytes after 48 h. This finding is in contrast to results obtained after 24 h at mRNA level and in staining for astrocytes incubated with 1 µg/mL LPS [45]. The 100-fold higher concentration, the shorter exposure time and the activation of other TLRs than TLR4 might explain the difference between studies. The absence of GFAP and further cytoskeletal proteins was reported to promote axonal sprouting and functional recovery in spinal cord injured animals. The comparison between wild type and GFAP null mice showed no differences in glial scar formation after injury [46,47]. Notably, formation of astrocytic processes was reduced when decreased GFAP expression was present. An increase in the expression of GFAP was correlated with reduced migratory capabilities [44,47]. Since NAGly is neuroprotective and astrocytes express GPR18 it seems plausible that at least part of protective actions of NAGly are mediated by astrocytes possibly via changes in GFAP expression followed by changes in the neuronal recovery and cellular migration to the lesion site. NAGly could exert the neuroprotective effect directly on neurons via GPR18, since CB_2_ expression is highly controversial [48,49] or NAGly mediated protection result from changes in all cell types, microglia, astrocytes and neurons.

The signaling mechanisms for NAGly and GPR18 have been explored in different models. NAGly mediated actions occurred amongst others via G_αi/o_ coupled receptors [5,7,9,50]. In ß-arrestin recruitment a NAGly mediated activation of GPR18 was missing [14] and some authors doubt that NAGly is a GPR18 ligand [8,13,14]. The activation of cannabinoid receptors in general is associated with particular signaling cascades, like Akt, p44/42 MAPK, JNK and others [51]. Here the phosphorylation of Akt in BV2 cells was reduced after 30 min incubation with NAGly. Akt is important in the regulation of cell survival and proliferation [52] and in microglia cells PI3K/Akt seems to play an anti-inflammatory role, if activated [53]. In human microglia the blockade of PI3K pathway had a stimulatory effect on proinflammatory gene expression [53]. The authors suggested that PI3K/Akt pathway is crucial for induction of anti-inflammatory and immunomodulatory microglia by suppressing proinflammatory and augmenting anti-inflammatory cytokine expression profiles. Since the species differences are present, it is difficult to directly compare NAGly mediated reduction in the activation of Akt in murine microglia. NAGly mediated protection may also be independent from Akt, since combinational treatment of LPS and NAGly had no effect. However, it is possible that NAGly mediated activation of GPR18 and following decrease in Akt expression can induce a “switch” of microglia to its proinflammatory phenotype like in Takenouchi et al. 2012 [5]. 

In our study no activation of p44/42, p38 MAPK and CREB in glial cells was observed. Conflicting results have been reported in earlier studies. Whereas McHugh et al. showed the phosphorylation of p44/42 for NAGly in stable transfected GPR18-HEK293 cells [54], Finlay et al. (2016) could not observe a cAMP modulation and failed to induce the p44/42 MAPK phosphorylation in HEK293-hGPR18 cells and in endogenously expressing GPR18 glioblastoma cells [8]. The p38, p44/42 MAPK and Janus kinases (JAK) pathways were activated and involved in apoptotic effects of much higher concentrations of NAGly (30 µM) in macrophage cell line RAW264.7 [5]. In GPR18 transfected HEK293 cells NAGly activated the p44/42 MAPK phosporylation 5 min post exposure [55]. Our study performed in BV2 cells and primary astrocytes supports the results obtained by Finlay and colleagues. Console-Bram postulated a biased agonism at GPR18, showing that the application of different ligands like NAGly, abnormal Cannabidiol, THC and O-1918 lead to initialization of different signaling cascades, possibly explaining the differences between studies [55]. 

## 4. Materials and Methods 

### 4.1. Ethics Statement

All animal experiments were performed in accordance with the Policy on Ethics and the Policy on the Use of Animals in Neuroscience Research as indicated in the directive 2010/63/EU of the European Parliament and of the Council of the European Union on the protection of animals used for scientific purposes and were approved by the local authorities for care and use of laboratory animals (State of Saxony-Anhalt, Germany, permission number: I11M18, date: 01.12.2012). 

### 4.2. Chemicals

*N*-arachidonoyl glycine (NAGly, Tocris Cookson, Bristol, UK), 1,3-Dimethoxy-5-methyl-2-[(1R,6R)-3-methyl-6-(1-methylethenyl)-2-cyclohexen-1-yl]benzene (O-1918, Tocris) and 6-Iodo-2-methyl-1-[2-(4-morpholinyl)ethyl]-1H-indol-3-yl](4-methoxyphenyl) methanone (AM630, Tocris) were dissolved in DMSO. Lipopolysaccharide (LPS, Sigma Aldrich, Munich, Germany), N-Methyl-D-Aspartate (NMDA, Sigma Aldrich) and adenosine triphosphate (ATP, Sigma Aldrich) were diluted in Aqua dest. All chemicals were stored at −20 °C or −80 °C until use. 

### 4.3. Organotypic Hippocampal Slice Cultures (OHSC)

108 C57BL/6J mice were sacrificed and used for experiments. To prepare OHSC, 5-day-old C57BL6/J mice were decapitated and their brains were dissected under aseptic conditions as published before [17,18,41]. The frontal pole and the cerebellum were removed and the brains were placed in minimal essential medium (MEM, Invitrogen, Carlsbad, CA, USA) containing 1% (*v*/*v*) glutamine (Invitrogen), 1% (*v*/*v*) penicillin/streptomycin (Invitrogen) and 1% (*v*/*v*) glucose (Sigma Aldrich, 45%) at 4 °C. 350 μm-thick OHSC were prepared using a sliding vibratome (Leica VT 1200, Leica Microsystems AG, Wetzlar, Germany).

Four to six OHSC were obtained from each brain and immediately transferred into cell culture inserts (pore size 0.4 μm, Millipore, Schwalbach/Ts., Germany) that were placed in 6-well culture dishes (Greiner, Frickenhausen, Germany) containing 1 mL culture medium per well. The culture medium consisted of 50% (*v*/*v*) MEM, 25% (*v*/*v*) Hanks’ balanced salt solutions (HBSS, Invitrogen), 25% (*v*/*v*) normal horse serum (NHS, Invitrogen), 2% (*v*/*v*) glutamine, 1 μg/mL insulin (Boehringer, Mannheim, Germany), 1.2 mg/mL glucose (Sigma Aldrich), 1% (*v*/*v*), streptomycin/penicillin (Invitrogen), and 8 μg/mL vitamin C (Sigma Aldrich), pH 7.4. The culture dishes were incubated at 35 °C in fully humidified atmosphere with 5% (*v*/*v*) CO_2_ and the cell culture medium was changed every second day.

### 4.4. Primary Cell Cultures and Cell Lines

Primary microglia and astrocytes were isolated from cerebral cortices of neonatal wild type mice as described before [56]. Briefly brains were treated with 4mg/mL trypsin (Merck Millipore, Burlington, MA, USA) and 0.5 mg/mL DNAse (Worthington, Bedford, MA, USA) in Hank’s balanced salts solution (Invitrogen). Murine primary cells and the murine microglial cell line BV2 and hippocampal neuronal cell line HT22 were cultured in DMEM (Invitrogen) with 10% FBS (Invitrogen) and 1 mL streptomycin/penicillin as described before [17,57]. 

Primary neurons were prepared as published before [57]. Dissociation of murine hippocampi was performed in papain rich medium at 37 °C and the cells were cultured on PLL coated glass coverslips for at least 10 days before the fixation with PFA or collecting for PCR.

BV2 cells [58] were obtained from Dr. Ullrich (University of Zürich) and HT22 cells [59,60] were obtained from Dr. Rami (Goethe University).

The preparations were divided into different groups and treated according to the following protocols (Figure 3). 

CTL: Unlesioned OHSC were kept in culture medium for 17 days in vitro (div) and served as controls. 

NAGly: At 14 div NAGly (10 µM) was added to the medium and kept until 17 div. 

NMDA: Another group of OHSC was lesioned with 10 μM NMDA at 14 div for 4 h and kept in culture medium for another 3 days.

NMDA + NAGly: NMDA lesioned OHSC were incubated in medium containing NAGly (0.1 µM, 1 µM, 10 µM) from 14 div till 17 div. 

NMDA + Antagonist + NAGly: Excitotoxically lesioned slices were incubated with an antagonist for GPR18, O-1918 (30 µM) or CB_2_, AM630 (10 µM) for 15 min, followed by incubation with a combination of NAGly and the respective antagonist until 17 div.

For time-dependent qRT-PCR and Western blot analyses two or three slices from unlesioned or lesioned groups (CTL or NMDA) were pooled and investigated at the time points 0 h, 30 min, 1 h, 2 h, 6 h, 12 h, 16 h, 24 h, 48 h and 72 h.

### 4.5. qRT-PCR

The collected OHSC were used for qRT-PCR analysis. Total RNA was isolated from slices using Trizol (PeqLab, Erlangen, Germany) following standard protocols [61]. The extracted RNA was re-suspended in nuclease-free water (Promega, Madison, WI, USA) and DNase treated (DNase Kit DNAFreeTM, Invitrogen). RNA yield was assessed by spectrophotometry. cDNA was synthesized from 5 μg of total RNA by reverse transcription using Transcription System (Promega).

PCR reactions were performed in a 17 μL reaction volume containing 10 µL PCR Mastermix (Promega), 0.25 µL Eva Green, 0.5 µL of each primer (Table 1), 4.75 µL DNase free water (Promega) and 1 μL cDNA template. Reactions were performed on a Rotor-Gene TM RG 6000 Thermocycler (Corbett Research, Pty Ltd., Sydney, Australia). In all cases the same amplification conditions were used: an initial denaturation step for 2 min at 95 °C, followed by 40 cycles of 94 °C for 30 s, 62–64 ° C for 30 s (mouse primer), 72 °C for 30–40 s, 2 min at 40 °C, 1 min at 50 °C with a measurement 50–99 °C during 1 °C/min. 

Correct amplicon size of PCR products was confirmed using 1.5% (*v*/*v*) agarose gels (PeqLab) containing GelRed™ Nucleic Acid Gel Stain (Biotium, Hayward, CA, USA) and visualized under UV light. To ascertain the quality of the first-strand cDNA and as loading controls, parallel PCR reactions were performed with species-specific primers (mouse) for the house-keeping gene ß-actin.

### 4.6. Western Blot Analysis

BV2 cells and astrocytes were seeded into 6 well plates (300,000 cells/well) and incubated in DMEM with 2% fetal bovine serum (2% DMEM) for 24 h prior to treatment. All stimuli were added into 2% DMEM. At the beginning of the experiment the medium was replaced with medium containing NAGly (0.1 µM), LPS (10 ng/mL) or ATP (100 µM) or combination of NAGly with LPS or ATP. For Western blot analysis cells were collected at time point 0 h and after 10 min, 30 min, 2 h, and 24 h respectively. The slices or cells were scraped in lysis buffer containing 80 mM Tris, 70 mM SDS, 0.3 M sucrose, 3 mM sodium orthovanadate and 0.5 mM phenylmethylsulfonyl fluoride (PMSF) at pH 7.4, collected and stored at −80 °C. Ten µg protein was loaded onto a 12.5% (*w*/*v*) sodium dodecyl sulfate–polyacrylamide gel. Proteins were electrotransferred to nitrocellulose membranes (Protran 0.45 µm, Amersham, Freiburg, Germany) and non-specific protein-binding sites were blocked for 30 min with Roti-block solution (Carl Roth, Karlsruhe, Germany). All antibodies (Table 2) were diluted in Roti block solution and washed with washing buffer between the incubation steps. The incubation with primary antibodies was performed overnight at 4 °C. On the next day membranes were washed and the secondary horse-radish peroxidase-conjugated antibodies were applied for 1 h. The signal of the bound antibody was visualized by enhanced chemiluminescence (Luminata Forte, Millipore, Schwalbach/Ts., Germany) with Fusion X. Image J image analysis software (version 1.46r, National Institutes of Health, Laboratory for Optical and Computational Instrumentation, University of Wisconsin, Madison, WI, USA) was used for semi-quantitative analysis of the intensity of the immunoreactive bands. 

### 4.7. Immunohistochemistry

One day prior to experiments, primary cultures of microglial cells, astrocytes, or BV2 cells were transferred into 24-well dishes coated with poly-L-lysine (Sigma Aldrich) and were stimulated up to 48 h with NAGly (0.1 µM, 10 µM) or LPS (10 ng/mL) or a combination of LPS and NAGly and fixed with 4% PFA. For immunocytochemistry cells were washed with 0.2 M phosphate buffered saline (PBS), and methanol with 3% H_2_O_2_ was used to block the endogenous peroxidase. After blockade of non-specific binding sites with normal goat serum, a primary antibody against GFAP (1:200, rabbit, BD Pharmingen, Heidelberg, Germany) or HRP-conjugated isolectin IB4 (1:50, Vector Laboratories) was applied overnight. Astrocytes were incubated with secondary biotinylated-anti-rabbit antibody (Sigma Aldrich) for 1 h, washed and afterwards Extra-Avidin-Peroxidase was applied (Sigma Aldrich). 3′,3′-Diaminobenzidine (DAB, Sigma Aldrich) was used for visualization. The pictures were recorded with a Zeiss microscope (Axioplan/Axiophot, Oberkochen, Germany). The ramification index (RI) was used as an indicator for activation of primary microglia by the so-called perimeter method as the ratio between the cell area and the corresponding territorial area. It is approximately 1 for amoeboid cells and tends to 0 for strongly ramified cells. The RI was evaluated using a MatLab (The MathWorks, Natick, MA, USA) script, the activation of astrocytes was calculated manually by the use of Image J. 

For double staining few OHSC were incubated after fixation with succhrose (Carl Roth, Karlsruhe, Germany) and cut on cryostat (Leica, Wetzlar, Germany) in 12 µm thick slices. 

OHSC, primary neurons, microglia and astrocytes were stained according to the same protocol. First normal goat serum was applied for 30 min before incubation with primary antibodies overnight (GFAP, 1:200, BD Biosciences, Franklin Lakes, NJ, USA or NeuN, 1:500, Merck Millipore). On the next day and after washing with PBS/Triton the incubation with GPR18 (1:300) antibody occurred. On the third day washing steps were performed, before application of secondary antibodies (donkey anti-mouse-Alexa 488, 1:200; goat anti-rabbit-Alexa 568, 1:200, Invitrogen) or fluorescein conjugated IB4 (1:50, Vector laboratories) for one hour, followed by incubation with DAPI (Sigma Aldrich). Finally, sections were washed and covered with DAKO mounting medium. Zeiss confocal laser-scanning microscope (CLSM, LSM 710 Meta, Zeiss, Göttingen, Germany) was used for documentation.

### 4.8. Propidium Iodide Staining and Confocal Laser Scanning Microscopy

Two hours prior to fixation with 4% paraformaldehyde (PFA, Sigma Aldrich) propidium iodide (PI, 5 µg/mL) was added to the culture medium. The uncut OHSC were removed from the cell culture inserts, washed with phosphate buffered saline (PBS) containing 0.03% (*v*/*v*) Triton X-100 (Applichem, Darmstadt, Germany; PBS-T) for 10 min, following 5 min with Aqua dest. and mounted with DAKO fluorescent mounting medium (DAKO Diagnostika GmbH, Hamburg, Germany).

Further analyses were done with a CLSM. For detection of PI labeled degenerating neurons and Alexa 568, monochromatic light with a wavelength of 543 nm and emission band pass filter for wavelength of 585–615 nm was used. For detection of Alexa 488 and DAPI following excitation wavelengths were used 488 nm and 405 nm, respectively. Emission was detected in the range of Δλ = 400–500 nm (DAPI), Δλ = 510–550 nm (Alexa 488). Double labeled slices and cells were assessed with 63× objective. 

Using the Z-mode of the CLSM, the PI—treated OHSC were optically cut into 2 μm thick sections. For counting PI positive neurons, the image was first denoised using a Wiener filter with a kernel size of 5 pixels. Afterwards a threshold was set manually and the image converted into a binary image. PI positive cells were than identified using the Hough transformation and measured. The number of PI positive neuronal nuclei was counted in the granule cell layer (GCL) of the dentate gyrus as reported previously [17,62].

### 4.9. Analysis of Cell Motility

For cell motility analysis isolated microglia (4000 cells) were placed into 6 well plates covered with poly-L-lysine 16 h prior to the start of experiments and treated with NAGly (0.1 µM, 10 µM) or ATP (100 µM) or both. The experiments and analysis were performed as explained elsewhere [41]. For studying single cell motion one frame per 5 min was taken with a 10× phase contrast objective and recorded with a CCD Camera (Leica) for 24 h. The obtained images were analyzed using a custom written MatLab script that determines the edge of the cell using a gradient operator and tracks each cell individually as published [41]. The mean speed of each cell and its contact area to the substrate were evaluated as random motion of single cells in a uniform culture medium (without treatment) measured.

### 4.10. Statistical Analysis

Data are presented as mean standard error of the mean (SEM) or standard deviation (SD for live cell imaging experiments, ramification index) of at least 3 independent experiments. Data were analyzed with ANOVA followed by the Dunnett’s or Bonferroni test for more than two groups or Students T-test for two groups only. Statistical analysis was conducted using GraphPad Prism (v5). Differences were considered significant at *p* ≤ 0.05.

## 5. Conclusions

In conclusion, we demonstrated that NAGly induces neuroprotection in the model of excitotoxically lesioned organotypic hippocampal slice cultures. It is unclear which target is responsible for NAGly mediated actions or whether GPR18 actions are cell type dependent. NAGly seems to interact with both receptors GPR18 and CB_2_, but the molecular mechanism remains elusive. NAGly does not activate the CB_1_ receptor, which makes it a promising substance without psychotropic side-effects. Targeting microglia and endogenous regulators of neuroinflammation might be a possible strategy for treatment of nervous system diseases. NAGly seems to target both microglia and astrocytes, and possibly neurons, but the impact of molecular mechanism must be further examined.

## Figures and Tables

**Table 1:** Primers.

Primer	Left	Right	Product Size	Sequence
ß-actin	ACTCCTACGTGGGCGACGAGG	CAGGTCCAGACGCAGGATGGC	389 bp	NM_007393
gpr18	TGAAGCCCAAGGTCAAGGAGAAGT	TTCATGAGGAAGGTGGTGAAGGCT	163 bp	NM_182806.1

**Table 2:** Antibodies (Western blot).

Antibody	Concentration	Company	Article Number	Antibody ID
ß-actin	1:5000	Cell Signaling, Boston, USA	3700	AB_2242334
pAkt	1:2000	Cell Signaling	9271	AB_329825
phospho-CREB (Ser133)	1:300	Merck Millipore	06-519	AB_310153
t-CREB clone E306	1:1000	Merck Millipore	04-218	AB_1586958
GAPDH (14C10)	1:1000	Cell Signaling	2188	AB_561053
GPR18	1:5000	Dr Ken Mackie’s Lab	Antibody is described in [9]	
phospho-p38 MAPK (Thr180/Tyr182)	1:2000	Cell Signaling	4511S	AB_2139682
t-p38 MAPK	1:2000	Cell Signaling	9212S	AB_330713
phospho-p44/42 MAPK (Erk1/2) (Thr202/Tyr204)	1:4000	Cell Signaling	9101	AB_331646
t-p44/42 MAPK (Erk1/2)	1:4000	Cell Signaling	9102	AB_330744
horse anti-mouse IgG, HRP conjugated	1:10000	Vector laboratories, Burlingame, USA	PI-2000	AB_2336177
goat anti-rabbit IgG, HRP conjugated	1:20000	Vector laboratories	PI-1000	AB_2336198

## Abbreviations

CB_1_	cannabinoid receptor 1
CB_2_	cannabinoid receptor 2
GFAP	glial fibrillary acidic protein
GPR18	G protein-coupled receptor 18
GPR55	G protein-coupled receptor 55
LPS	lipopolysaccharide
MAPK	Mitogen-activated protein kinase
NAGly	N-arachidonoyl glycine
NMDA	N-methyl-D-aspartate receptor
OHSC	organotypic hippocampal slice cultures
SEM	standard error of the mean
SD	standard deviation
THC	Δ9-tetrahydrocannabinol
